# Gorham-Stout syndrome affecting the left mandible: A case report

**DOI:** 10.3892/etm.2012.793

**Published:** 2012-11-02

**Authors:** QINGSHAN DONG, YAFEI ZHANG, CHUANKONG SUN, JIAPING GUO

**Affiliations:** 1 Departments of Stomatology and; 2 Oncology, Wuhan General Hospital of Guangzhou Command, People’s Liberation Army, Wuhan 430070, P.R. China

**Keywords:** Gorham-Stout syndrome, osteolysis, left mandible

## Abstract

Gorham-Stout syndrome is an extremely rare condition in which spontaneous, progressive resorption of bone occurs. Owing to its low incidence and variable clinical presentation, the diagnosis is often missed or delayed, and at present, there are no specific guidelines for its treatment. We present the case of a 20-year-old male diagnosed with Gorham-Stout syndrome with involvement of the left mandible, and discuss its diagnostic and therapeutic features.

## Introduction

Gorham-Stout syndrome is a rare skeletal disorder, the etiology and pathogenesis of which remain unknown. Since the first description by Jackson *et al* in 1838, no more than 200 cases have been reported (1,2). The therapeutic procedure remains controversial owing to the rarity and progressive osteolysis of this disease, while reconstructive treatments are used in certain cases in an attempt to recover the function of the bone involved. According to clinical records, Gorham-Stout syndrome is usually initiated in a single bone (or very few bones) or contiguous bones around one focus (1,2). The maxillofacial skeleton is one region frequently affected. The first maxillofacial case was described by Romer *et al* in 1928 (3). Since then, ∼50 maxillofacial cases have been reported (4). In the present study, a case of Gorham-Stout syndrome affecting the left mandible in a 20-year-old male is presented.

## Case report

**Figure 1:**
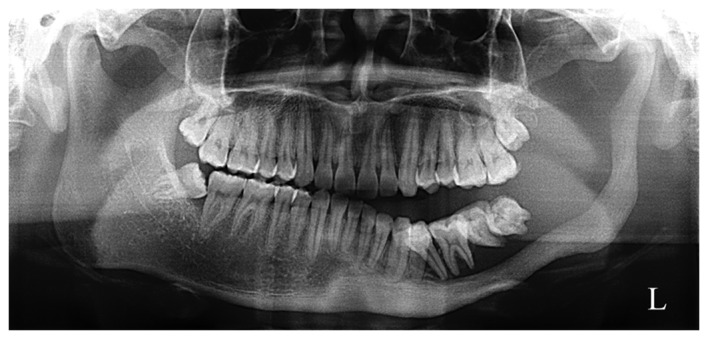
Panoramic radiograph revealing severe bone resorption of the left mandibular body and ramus.

**Figure 2:**
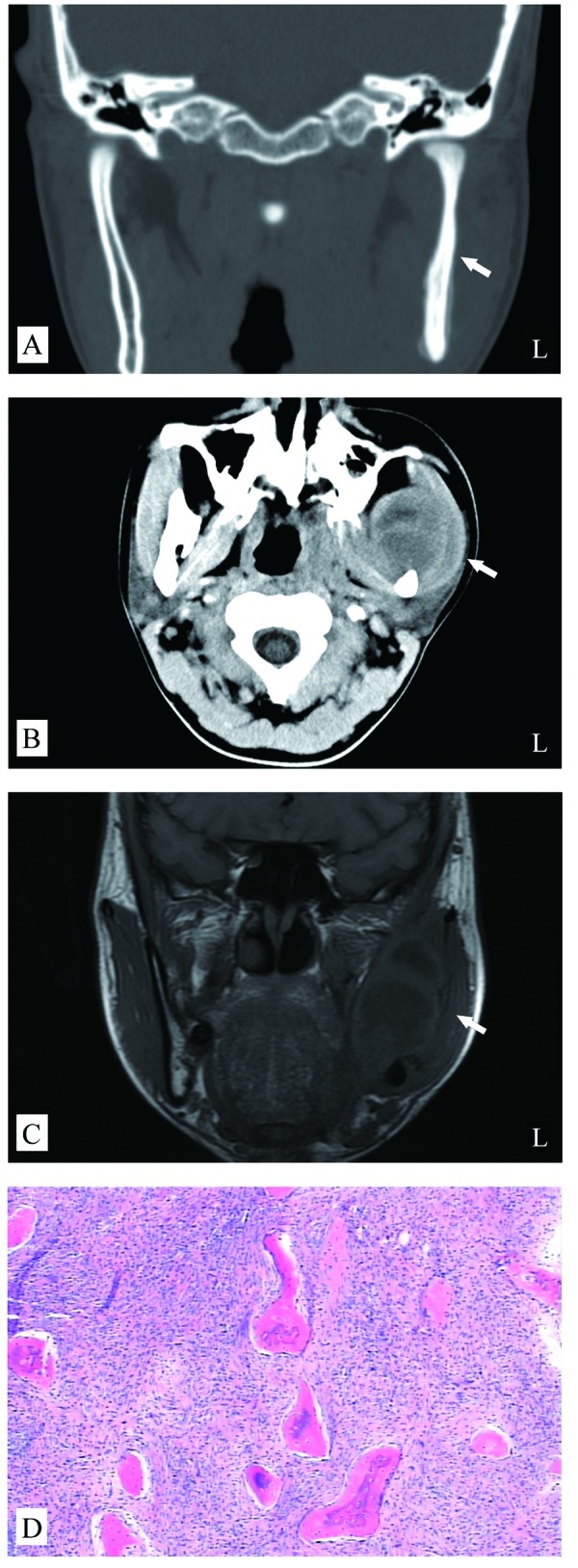
CT images of the maxillofacial region. (A) Coronal CT scan. Arrow shows that the left mandible bone has become thin, with incomplete sclerosis of the medullary cavity. (B) Axial CT scan. Arrow shows an irregular, thick-walled cystic mass in the left ramus of the mandible. (C) Coronal MRI scan. Arrow shows an irregular, thick-walled cystic mass in the left ramus of the mandible. (D) Histopathological examination of a bone biopsy. H&E ×100.

**Figure 3:**
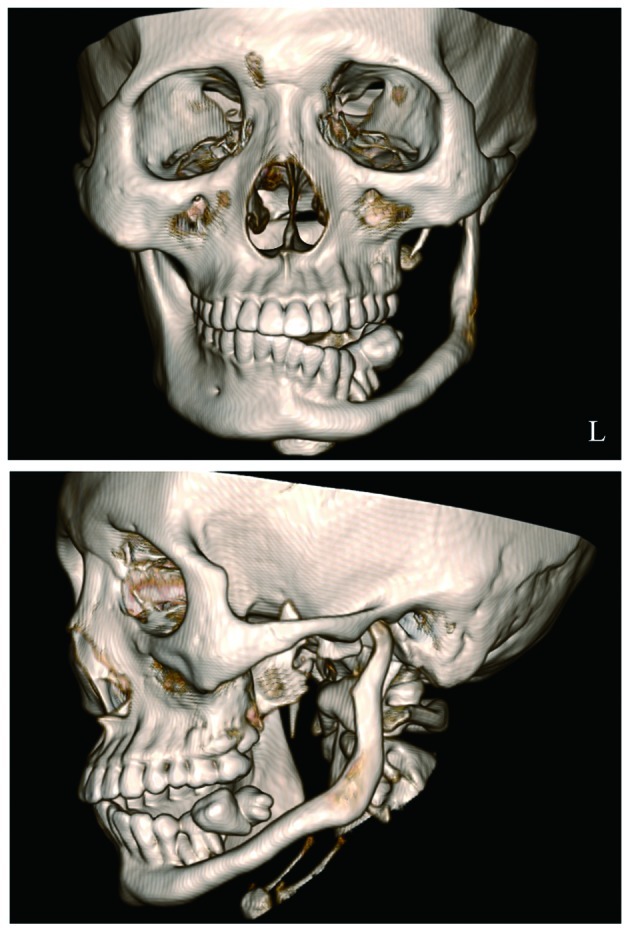
Three-dimensional reconstruction CT images of the maxillofacial region. Upper panel, front-view; Lower panel, lateral-view.

A 20-year-old male patient presented with a 6-year history of dental pain and progressive loosening of the posterior teeth on the left mandible. A panoramic radiograph revealed wide-ranging dissolution of the left mandible, while no periosteal reaction or reactive new bone formation were observed around the residual bone tissues (Fig. 1). A CT scan of the craniofacial region corroborated X-ray results, and further revealed that the osteolytic bone ranged from the left ramus to the second premolar, with a continuation of the lower margin of the left mandible only. The teeth appeared to float in the osteolytic tissues (Fig. 2A). Three-dimensional reconstruction CT images indicated that the left temporomandibular joint remained uninvolved (Fig. 3).

An initial diagnosis of pericoronitis of the wisdom tooth was issued by the patient’s local primary hospital. Following anti-inflammatory therapy, the dental pain was temporarily relieved but the progressive tooth loosening was also aggravated. In 2009, the chewing function in the left mandibular area was completely lost. Dental radiology at that time indicated massive osteolysis of the left mandible, however, the patient refused further examinations and therapy.

In May 2012, the patient was referred to our hospital due to an enlarging mass in the left mandible. CT and MRI images showed a 38×43×75-mm, irregular, thick-walled cystic mass in the left ramus of the mandible (Fig. 2B and C). Standard laboratory investigations, including bone metabolism tests, revealed no abnormality. A skeletal survey revealed no other osseous involvement. The loosening posterior teeth (4–8) were extracted during exploratory surgery and a large quantity of pus was spilled from the incision area. Following repeated rinsing with sterile saline, the wound was sutured. Histopathological examination of the bone biopsy showed proliferation of the fibrous connective tissue, intermixed with irregular bony trabeculae (Fig. 2D). Tissues obtained from the cystic wall were also sent for histopathological examination, but only inflammation and granulation tissue formation were revealed.

On the basis of the clinical, radiographic and histopathological results, a diagnosis of Gorham-Stout syndrome was made. Radiotherapy and etidronate therapy were proposed to the patient, but were not accepted. At present, the patient remains under observation. The study was approved by the ethics committee of Wuhan General Hospital of Guangzhou Command. Written informed patient consent was obtained from the patient.

## Discussion

Numerous studies concerning the etiopathology and clinical presentation of Gorham-Stout syndrome have been reported, along with radiographic findings and therapeutic options, in order to raise the awareness of this rare disease.

Debates over the presence of osteoclasts in Gorham-Stout syndrome reveal the uncertainty of researchers about the exact etiopathology of the condition. Certain researchers consider that angiomatosis of the blood vessels and occasionally of the lymphatics is responsible (3), while others consider that a previous trauma must be involved (5). However, a definite etiology has not been established thus far.

The clinical presentation of Gorham-Stout syndrome is variable depending on the affected sites. Certain patients have presented with a relatively abrupt onset of pain and swelling or a pathological fracture on the affected site, whereas others have presented with a history of an insidious onset of pain, limitation of motion and progressive weakness in the affected area (3). The disease is not usually accompanied by any systemic symptoms (6). In most cases, the bone resorption process may stop spontaneously, and therefore the prognosis is generally good unless vital structures are involved.

A final diagnosis of Gorham-Stout syndrome is difficult. Laboratory findings are not specific and are of no value in the diagnostic procedure. Radiographs provide the most significant clues for obtaining a diagnosis. In early X-rays, Johnson and McClure (7) noted evidence of one or multiple centromedullary and subcortical radioluciencies, usually with indistinct margins and no sclerotic borders. Later, these lesions may enlarge and fuse together, causing a disruption of the cortex and then intraosseous and extraosseous resorption (7). CT scanning and three-dimensional reconstruction are more useful for accurately assessing the range of bone destruction at the time of diagnosis. MRI is used to define the extent of vascular formation and the involvement of the adjacent soft tissue. The histological findings depend on the phase in which the disease is diagnosed. In the first of the two phases, the bone-displacing fibrous tissue section exhibits a higher concentration of blood vessels, whereas only fibrous tissue is detected in the second phase (5,8). Hereditary and essential osteolysis, tumours, skeletal angiomas, infection and other causes of osteolysis should all be ruled out before a differential diagnosis of Gorham-Stout is made (9). Notably, the cystic mass in the present study emerged at the beginning of 2012, which is later than the appearance of the osteolysis of the left mandible. Therefore, it may be considered as a complication of Gorham-Stout syndrome.

Due to the rarity of this disease, there is no standard therapy available. The treatment modalities include surgery, radiotherapy, etidronate therapy and the use of α-2b interferon (1,3). In the present study, as there has been no marked progression in mandibular resorption since 2009, the patient and his family have refused any offer of further treatment and the patient’s condition is being observed via clinical follow-up.

Taking all the evidence together, considering the history and clinical manifestations and the radiographic and histopathological results, the diagnosis of Gorham-Stout syndrome in this case was considered to be reasonable and logical.
